# Effects of Bonding Parameters on Free Air Ball Properties and Bonded Strength of Ag-10Au-3.6Pd Alloy Bonding Wire

**DOI:** 10.3390/mi11080777

**Published:** 2020-08-14

**Authors:** Jun Cao, Junchao Zhang, John Persic, Kexing Song

**Affiliations:** 1 School of Mechanical and Power Engineering, Henan Polytechnic University, Jiaozuo 454000, China; caolinc@163.com; 2 Microbonds Inc, 7495 Birchmount Rd., Markham 60428, ON L3R 5G2, Canada; jpersic@microbonds.com; 3 School of Materials Science and Engineering, Henan University of Science and Technology, Luoyang 471000, China; kxsong@haust.edu.cn

**Keywords:** EFO current, EFO time, bonding power, bonding force, bonded strength

## Abstract

Free air ball (FAB) and bonded strength were performed on an Ag-10Au-3.6Pd alloy bonding wire (diameter of 0.025 mm) for different electronic flame-off (EFO) currents, times and bonding parameters. The effects of the EFO and bonding parameters on the characteristics of the FAB as well as the bonded strength were investigated using scanning electron microscopy. The results showed that, for a constant EFO time, the FAB of the Ag-10Au-3.6Pd alloy bonding wire transitioned from a pointed defined ball to an oval one, then to a perfectly shaped one, and finally to a golf ball with an increase in the EFO current. On the other hand, when the EFO current was constant and the EFO time was increased, the FAB changed from a small ball to a perfect one, then to a large one, and finally to a golf ball. The FAB exhibited the optimal geometry at an EFO current of 0.030 A and EFO time of 0.8 ms. Further, in the case of the Ag-10Au-3.6Pd alloy bonding wire, for an EFO current of 0.030 A, the FAB diameter exhibited a nonlinear relationship with the EFO time, which could be expressed by a quadratic function. Finally, the bonded strength decreased when the bonding power and force were excessively high, causing the ball bond to overflow. This led to the formation of neck cracks and decrease in the bonded strength. On the other hand, the bonded strength was insufficiently when the bonding power and force were small. The bonded strength was of the desired level when the bonding power and force were 70 mW and 0.60 N (for the ball bonded) and 95 mW and 0.85 N (for the wedge bonded), respectively.

## 1. Introduction

With the vigorous development of the information age, the electronic information industry has become an extremely important part of today’s society. Electronic products have been widely used in all aspects of social life, and promote the continuous development and technological innovation of the entire electronic industry [1,2]. The electronic and physical functions of semiconductors and electronic devices can be transformed into the form suitable for machine systems and serve human society [3]. Electronic packaging technology and bonding materials directly affect the quality and service life of electronic products, so it is necessary to study the packing process. Wire bonding is a kind of electronic interconnection technology which uses fine metal wire to provide connection capability by heat, pressure and ultrasonic energy [4,5]. Wire bonding has the advantages of simple process, low cost and suitable for a variety of packaging forms, wire bonding is the most widely used interconnection technology in microelectronic packaging [6].

Among the costs required for all IC products, the cost proportion of packaging has become increasingly high recently. In recent years, this figure has even reached 40%. At the same time, more than 25% of the failure rate of IC products is caused by package failure [7,8]. Nowdays, there are many kinds of bonding materials, among which the silver base alloy bonding wires exhibit excellent mechanical properties, good oxidation resistance, and high reliability and are cost effective. As a result, they can limit light attenuation and improve the conversion rate in light-emitting diode (LED) packaging. Because of these advantages, these wires are employed widely in integrated circuit and LED packaging [9,10,11,12,13,14,15,16].

In microelectronic packaging, the chip and the outside world can achieve smooth input and output through the connection of external pins and integrated circuits, which is the core of the whole microelectronic packaging process [17,18]. The factors that may affect the bonding quality are bonding time, bonding power, bonding force and temperature [19,20]. With respect to the wire bonding process, the shape of the free air ball (FAB) has a determining effect on the strength of the joint formed between the lead and the substrate. The bonding wire melts under the action of the electronic flame-off (EFO), and the FAB is formed because of the surface tension and gravity. The size of the FAB is small (the diameter is approximately 35–55 μm), and it solidifies rapidly. The surface tension and solidification characteristics of the FAB during the melting process determine its shape [21,22]. Once the FAB is formed, it is bonded to the aluminum substrate of the chip, with the bonding parameters such as the bonding pressure, ultrasonic power, and bonding time, determining the bonding characteristics. A diffusion layer (of an intermetallic compound) is formed because of surface and dislocation diffusion, and it also affects the bonded strength [23]. The geometry of the FAB determines the initial bond strength of the ball joint. Further, the sphericity and roundness of the FAB directly affects the bonded strength between the chip and the substrate, which is the primary factor affecting the strength of the ball bonded. The composition of the Ag-10Au-3.6Pd alloy is more complex than that of pure metal. As a result, the geometry of the FAB is affected to a greater degree by the EFO parameters. On the other hand, in the case of the Ag-10Au-3.6Pd alloy bonding wire, the bonded strength, which determines the device life, is also affected by the bonding power and force. It is also the future development trend to optimize the bonding process parameters to improve the wire bonding quality. In recent years, there have been several experimental studies on the ball-forming properties of alloy wires. Chen et al. [24] reported that the EFO current and time are the two most important parameters related to the balling process. Tan et al. [25] performed an experimental study on the burning of copper balls of different diameters and determined the empirical formula for describing the diameter of the copper ball based on the ball-burning current and time. Xu et al. [26] studied the effects of the ultrasonic power and bonding pressure on the bonded strength during bonding using Au wire and concluded that a mismatch between the ultrasonic power and bonding pressure can significantly reduce the bonded strength.

However, the above-described studies were mostly performed on wires of pure metals such as Cu and Au, and there has been little research on the effects of the ball-burning parameters on the ball-forming properties and bonded strength of the Ag-10Au-3.6Pd alloy bonding wire. In this study, the geometry of the FAB and the bonded strength of the Ag-10Au-3.6Pd alloy bonding wire was investigated for different EFO currents and times and bonding powers and forces, and the optimal bonding parameters for the wire were determined. The results of this study should serve as the theoretical basis for the use of the Ag-10Au-3.6Pd alloy bonding wire in microelectronic packaging.

## 2. Materials and Methods

**Figure 1:**
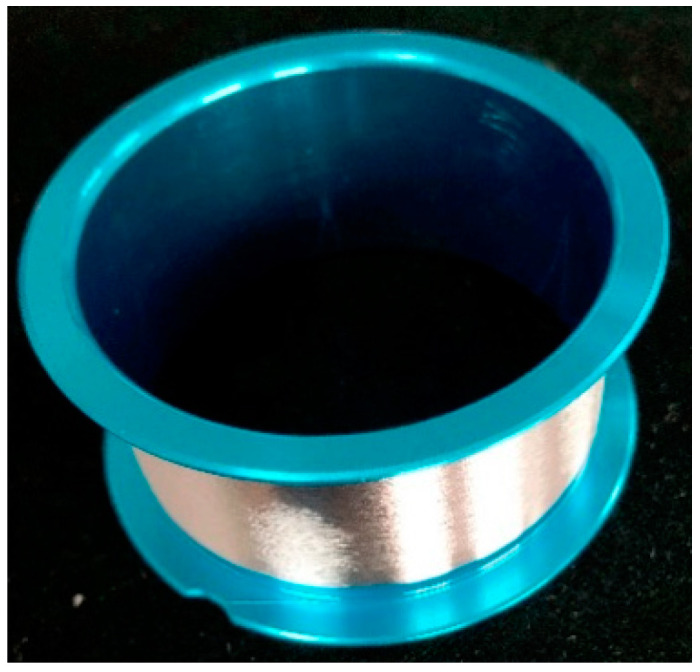
Ag-10Au-3.6Pd alloy bonding wire.

An Ag-10Au-3.6Pd alloy bonding wire with a diameter of 0.025 mm was employed in this study, shown in Figure 1. The wire had average elongation of 16.4% and average tensile strength of 0.102 N (broken load).

**Figure 2:**
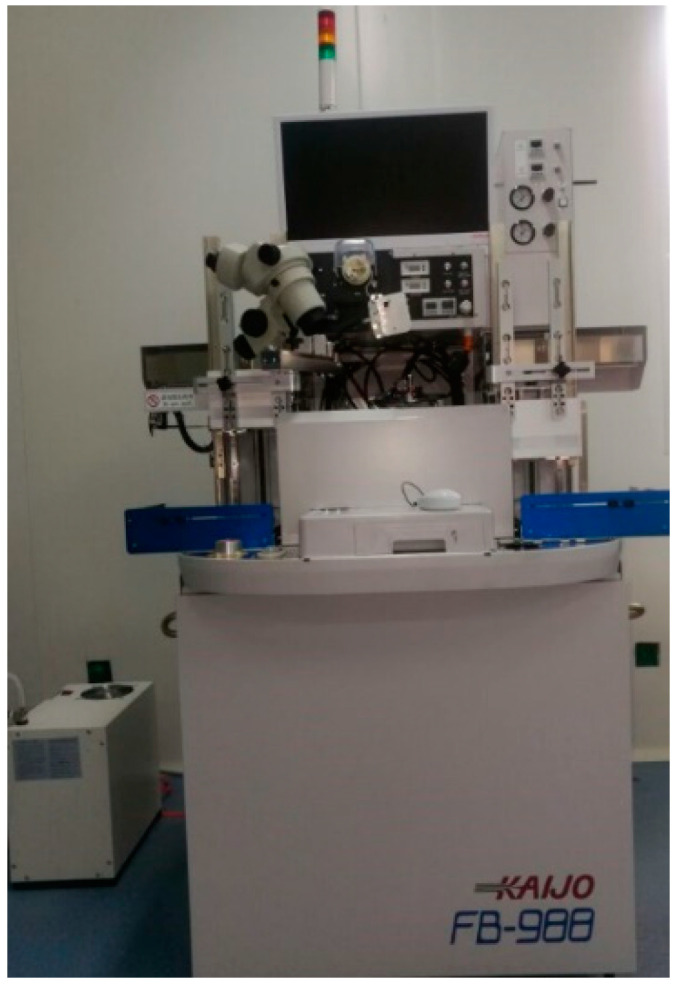
KAIJO FB-988 automatic wire bonder machine.

A KAIJO FB-988 automatic wire bonder (KAIJO Co., LTD, Tokyo, Japan) was used for the wire bonding process, shown in Figure 2. A packaging type 2835 LED was employed, and the capillary type used was SPT SU-50140-425E-ZU36-E. The bonding parameters are listed in Table 1. During the bonding process, a mixture consisting of 95 vol% N_2_ + 5 vol% H_2_ was used as the forming gas at a flow rate of 0.6 L/min to protect the wire from oxidation. The geometry and size of the FAB and the bond geometry of the bonding wire (diameter of 0.025 mm) were investigated using a scanning electron microscopy (SEM) system, and the relationship between the EFO time and FAB diameter was determined. The bonded strength (ball shear/pull strength and wedge pull strength) were measured using a DAGE Series 4000 BS250 system at a test speed of 500 μm/s and shear height of 5 μm.

## 3. Results

### 3.1. Effects of EFO Current on Geometry of FAB of Ag-10Au-3.6Pd Alloy Bonding Wire

**Figure 3:**
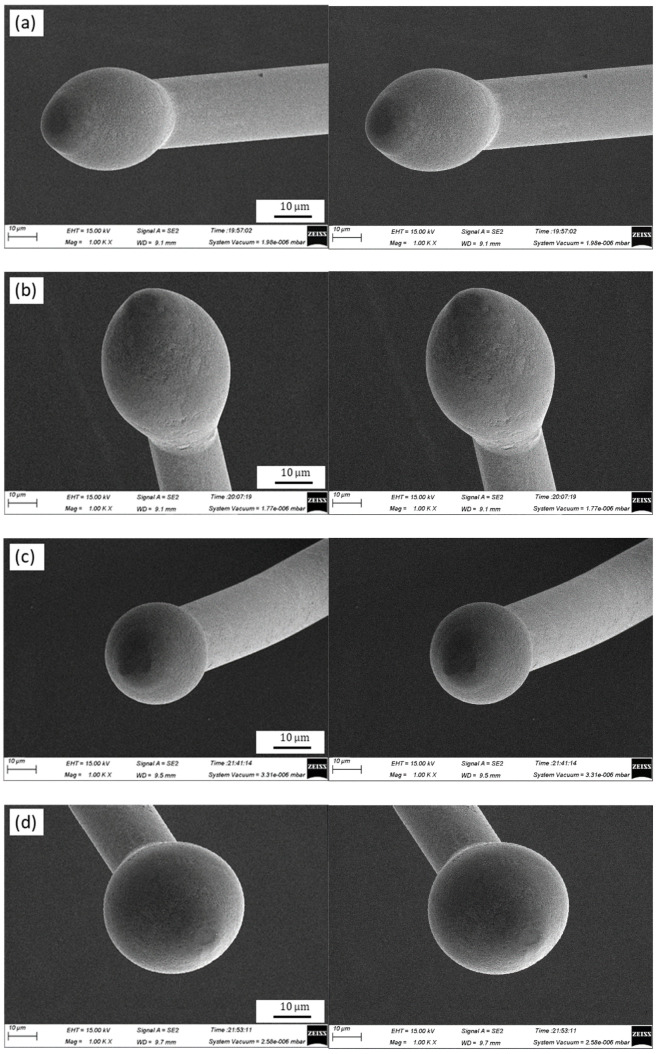

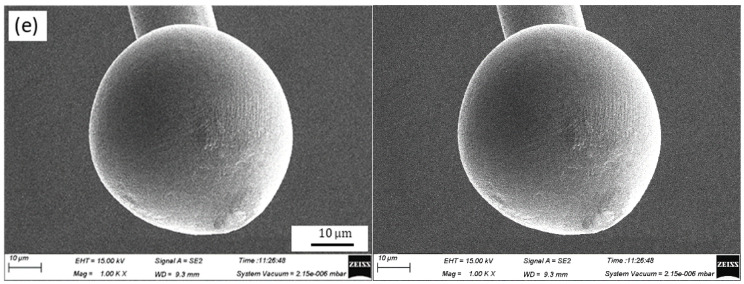
Geometries of FAB of Ag-10Au-3.6Pd alloy bonding wire for different EFO currents: (**a**) 0.015 A, (**b**) 0.020 A, (**c**) 0.025 A, (**d**) 0.030 A, and (**e**) 0.035 A.

The FAB geometries of the Ag-10Au-3.6Pd alloy bonding wire for different EFO currents are shown in Figure 3, the EFO time was held constant at 0.8 ms. Figure 3a shows that the FAB of the bonding wire was sharp and pointed when the EFO current was 0.015 A. Further, Figure 3b shows that the geometry of the FAB changed to oval when the EFO current was increased to 0.020 A. Moreover, it can be seen from Figure 3c that the FAB became round when the EFO current was increased further to 0.025 A. Next, Figure 3d shows that the FAB geometry did not change when the EFO current was increased from 0.025 A to 0.030 A; however, its diameter did increase. Finally, Figure 3e shows that the FAB became similar to a golf ball when the EFO current was 0.035 A. Further, the FAB was irregular, and this shape could potentially lead to short circuits.

The bonding wire melts and forms an FAB because of the surface tension of the molten metal after the EFO process. As the FAB solidifies, heat is transferred from un-melted bonding wire to its neck (i.e., the heat-affected zone). Hence, the FAB solidifies from its neck to its bottom, and its diameter is 2–3 times that of the wire. During the EFO process, a high voltage is generated between the EFO tip and the wire tail, because of which the air within the gap break downs, resulting in the transfer of energy to the wire tail, which forms the FAB. After the EFO process, the temperature of the FAB is higher than the melting point of the wire. As a result, the wire tail melts further, leading to the growth of the FAB. If the EFO current is too small (0.015 or 0.020 A), the FAB bottom solidifies because of a lack of energy, and the solidification sequence is broken, resulting in a pointed and subsequently oval ball, as shown in Figure 3a,b, respectively. However, when the EFO current is increased from 0.020 A to 0.025 A, more heat is produced because of the higher EFO current, and the wire tail melts completely. Consequently, the FAB solidifies from its neck to its bottom. Thus, a regular FAB is formed under the surface tension, as shown in Figure 3c. When the EFO current is increased further to 0.035 A, the wire tail melts, and the FAB is formed rapidly because of the large current. Further, the volume of the FAB is larger. Conversely, when the part of the wire tail closest to the EFO tip melts faster than that away from the tip, it causes the center of the FAB to deviate from that of the wire such that the former is closer to the EFO tip side. As a result, a golf ball FAB is formed, as shown in Figure 3e.

### 3.2. Effects of EFO Time on Geometry of FAB of Ag-10Au-3.6Pd Alloy Bonding Wire

**Figure 4:**
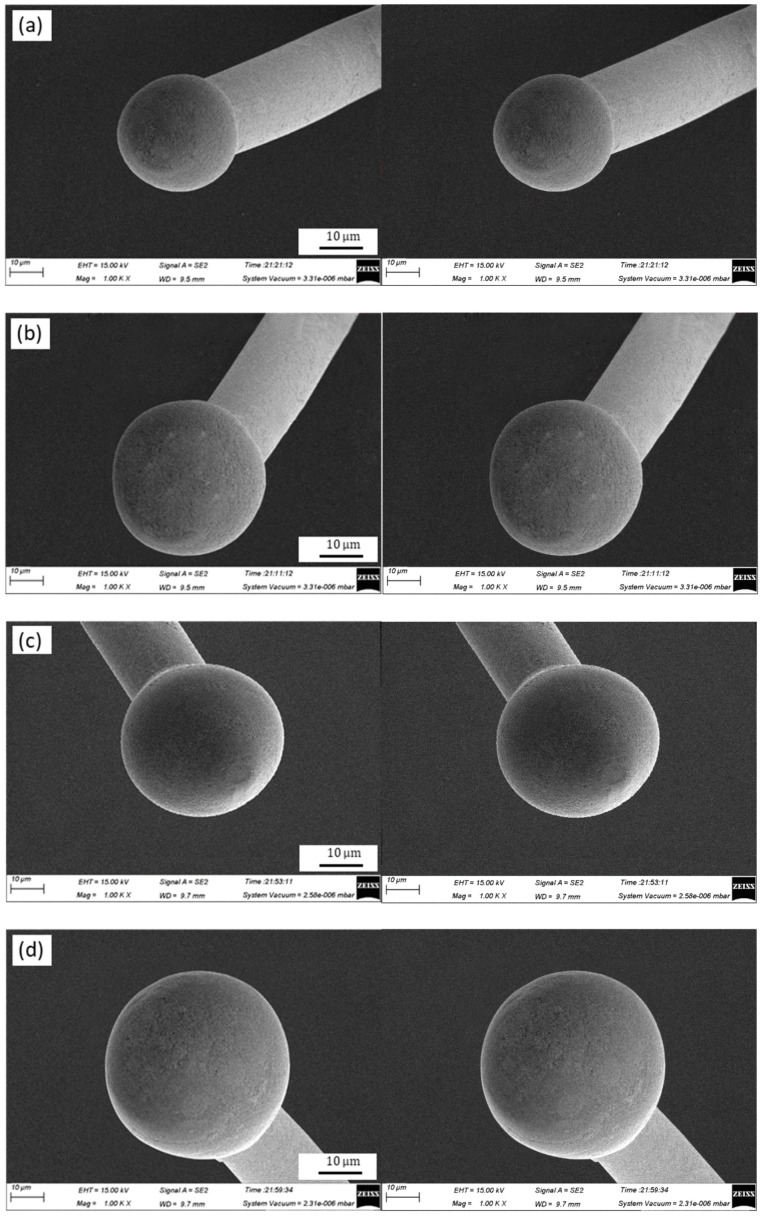

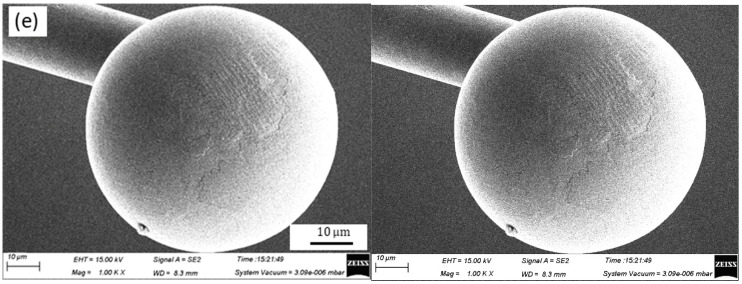
Geometries of FAB of Ag-10Au-3.6Pd alloy bonding wire for different EFO times: (**a**) 0.4 ms, (**b**) 0.6 ms, (**c**) 0.8 ms, (**d**) 1.0 ms, and (**e**) 1.2 ms.

The geometries of the FAB of the Ag-10Au-3.6Pd alloy bonding wire for different EFO times are shown in Figure 4, the EFO current was held constant at 0.030 A. Figure 4 shows that the FAB is a regular ball when the EFO time is 0.40 ms, however, the diameter of the FAB is smaller than that of a standard FAB, as shown in Figure 4a. The FAB continues to be a regular ball when the EFO time is increased from 0.40 ms to 0.60 ms, however, the FAB diameter increases, as shown in Figure 4b. The FAB geometry becomes that of a standard ball when the EFO time is increased to 0.80 ms, as shown in Figure 4c. However, the FAB geometry does not change when the EFO current is increased further from 0.80 ms to 1.00 ms. On the other hand, the FAB diameter increases, as shown in Figure 4d. Finally, the FAB becomes a golf ball when the EFO time is increased to 1.20 ms, as shown in Figure 4e.

**Figure 5:**
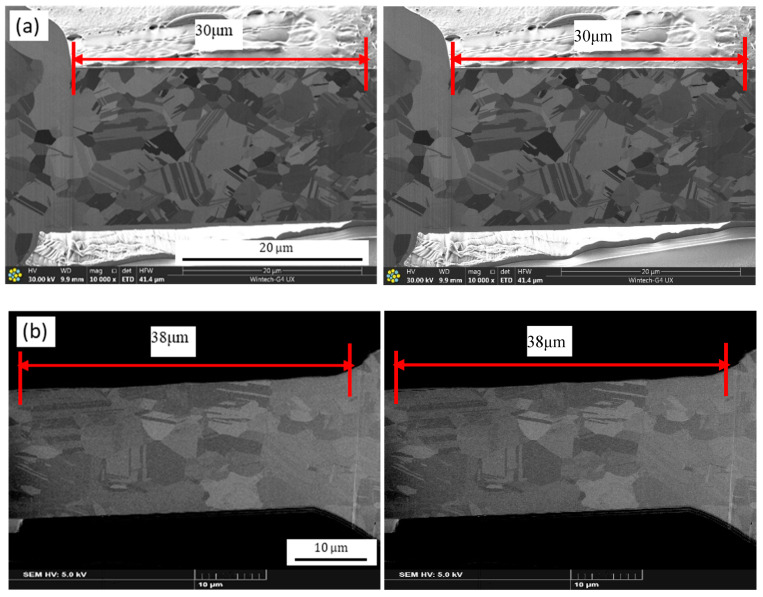
Length of heat-affected zone of Ag-10Au-3.6Pd alloy bonding wire for different EFO times: (**a**) 0.8 ms and (**b**) 1.0 ms.

Figure 4a,b show that, for a fixed EFO current of 0.030 A, the EFO tip produces enough energy to melt the wire tail, such that the length of the melted wire tail becomes shorter as the EFO time is reduced to 0.40 and 0.60 ms, resulting in a smaller FAB. However, the FAB is round and regular. As can be seen from Figure 4c, the FAB becomes a standard ball when the EFO time is increased to 0.80 ms. Further, Figure 4d shows that the wire tail melts more when the EFO time is increased to 1.00 ms; the diameter of the FAB also increases. However, the FAB center remains unchanged. When the EFO current is kept constant (at 0.030 A), the EFO tip produces the same amount of energy irrespective of the time. As a result, the FAB grows in a regular process from the wire tail tip to the capillary and exhibits a regular geometry. Moreover, its diameter is larger than that in the case of an EFO time of 0.80 ms. On the other hand, a larger FAB result in an excessively large ball bonded, with the probability of a short circuit occurring due to the bonded ball increasing. Further, the bonding efficiency decreases when the EFO time is increased. When the EFO time is increased from 1.00 ms to 1.20 ms, the FAB volume increases further, and the large FAB solidifies from its neck to its bottom, with heat being transferred along this path. The temperature distribution of the FAB will be uneven because of its large diameter, and the part of the FAB near its neck will solidify first. The other parts solidify in an upward direction because of surface tension, resulting in the FAB exhibiting the geometry of a corrugated ball. In addition, the length of the heat-affected zone of the wire neck increases owing to the transfer of a greater amount of heat from the ball to the wire. For instance, the length increases from 30 μm to 38 μm when the EFO time is increased from 0.80 ms to 1.00 ms, as shown in Figure 5a,b. This reduces the neck strength. Moreover, the melt rate of the wire tail closer to the EFO tip side is higher than that for the opposite side, owing to the excessively long EFO time. The extent of melting of the wire tail closer to the EFO tip side is higher, causing the FAB center to deviate from that of the alloy wire. This results in the FAB becoming a golf ball, as shown in Figure 4e.

### 3.3. Relationship between Diameter of FAB of Ag-10Au-3.6Pd and EFO Time

For alloy bonding wire, at the moment when electronic flame off (EFO) is generated, the surrounding air is ionized, and the alloy wire at the end melts, forming a metal melt free air ball (FAB) under the combined action of gravity and surface tension. The higher EFO power and the longer EFO time, the bonding wire absorbs more energy and the larger FAB diameter formed. However, if the parameters are too large, the FAB size will be too large, and the neck strength of joint formed by bonding will be too small, which will lead to the fracture of joint neck. In addition, the EFO power and EFO time should be matched with each other. When the EFO power is increased, the EFO time should be appropriately prolonged to fully absorb the energy and improve the bonding quality [26].

During the wire bonding process, the FAB geometry and diameter are the primary factors determining the strength of the ball bonded. A large FAB diameter is tolerated in the case of large die pad chips, as the bonded area increases with the FAB diameter, resulting in a higher bonded strength. However, for smaller die pad chips, an excessively large FAB can cause the bonded ball to overflow, resulting in an increase in the potential for short circuits.

**Figure 6:**
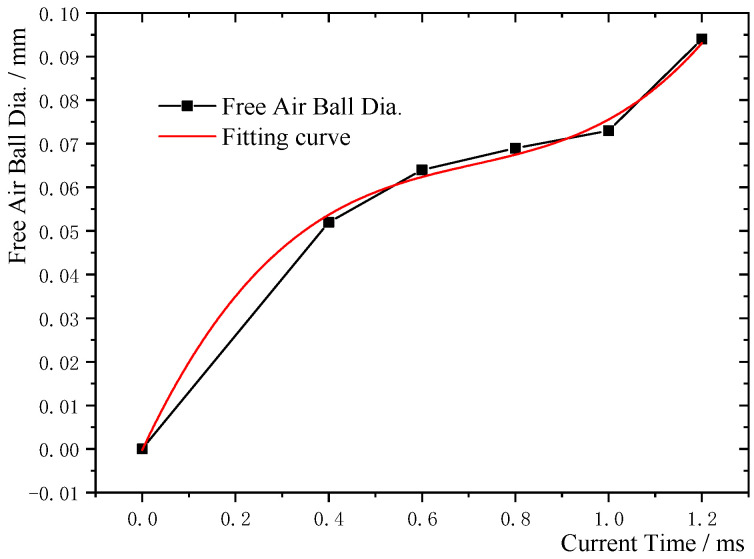
Fitted curve for relationship between FAB diameter (*D*) and EFO time (*t*) for Ag-10Au-3.6Pd alloy bonding wire with diameter of 0.025 mm.

The experimental data for the FAB diameter and EFO current time for a wire diameter of 0.025 mm were fitted, the results are shown in Figure 6 (the EFO current was set at 0.030 A). It can be seen from the figure that the FAB diameter and EFO time exhibited an nonlinear relationship. Using the least-squares method for data fitting, the following quadratic equation was obtained for the relationship between the FAB diameter and EFO time:

(1) $$D=-2.61905\times 10^{-4}+0.22851-0.2881t^{2}+0.13542t^{3}$$

where *D* is the FAB diameter (mm) and *t* is the EFO time (ms).

Further, using the least-squares analysis method, the correlation coefficient, *r*, between the two variables was calculated and found to be 0.99698, with the confidence interval being 0 < *t* < 1.20. Thus, it was confirmed that the obtained equation was a reasonable one and thus can be used by the packaging industry for determining the EFO time for Ag-10Au-3.6Pd alloy bonding wires with a diameter of 0.025 mm.

### 3.4. Effects of Bonding Power and Force on Ball Bonded Strength of Ag-10Au-3.6Pd Alloy Wire

**Figure 7:**
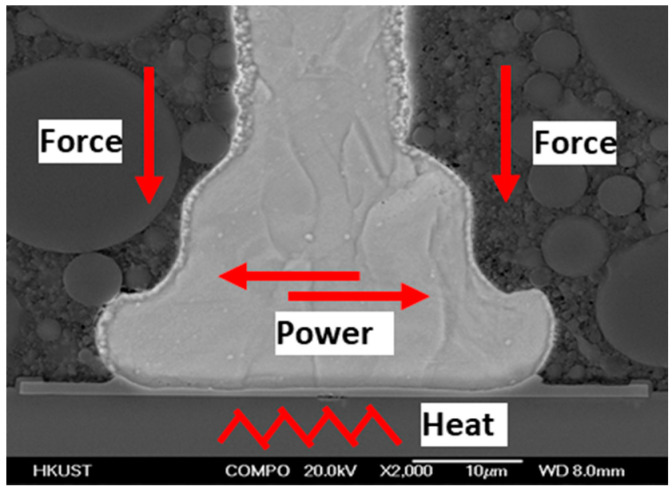
Schematic of ball bonded.

**Figure 8:**
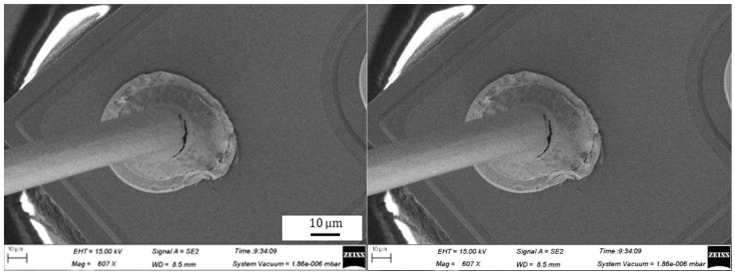
Geometry of ball bonded for bonding power of 85 mW and bonding force of 0.75 N.

**Figure 9:**
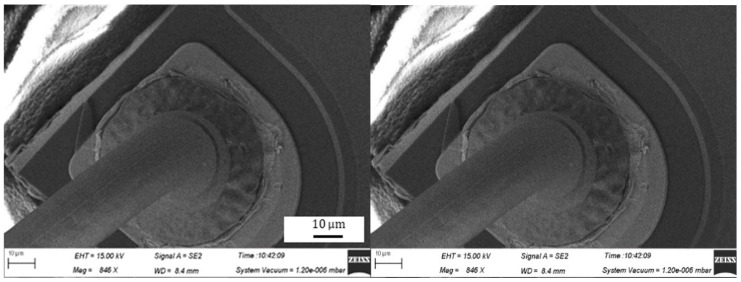
Geometry of ball bonded for bonding power of 70 mW and bonding force of 0.60 N.

**Figure 10:**
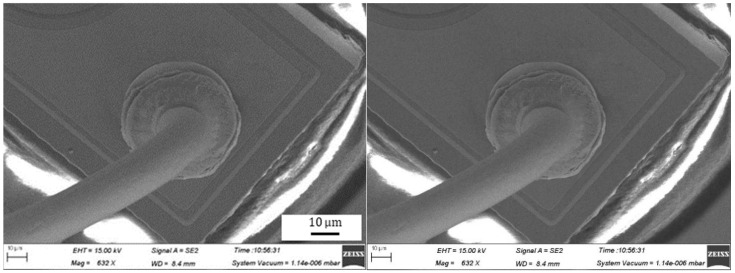
Geometry of ball bonded for bonding power of 55 mW and bonding force of 0.45 N.

**Figure 11:**
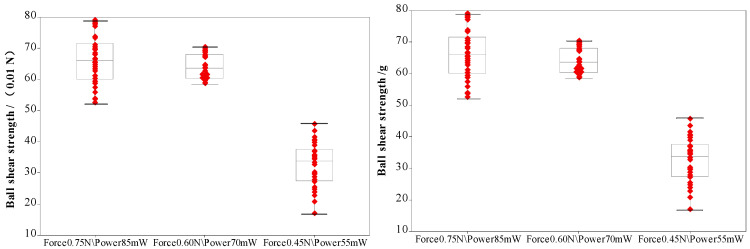
Shear strength of ball bonded.

**Figure 12:**
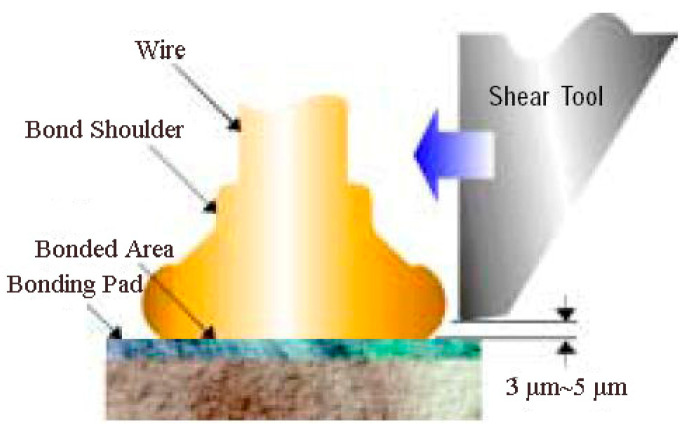
Mechanism of ball shear strength testing [27].

**Figure 13:**
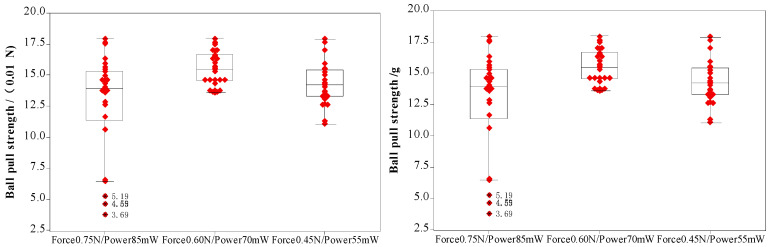
Pull strength of ball bonded.

**Figure 14:**
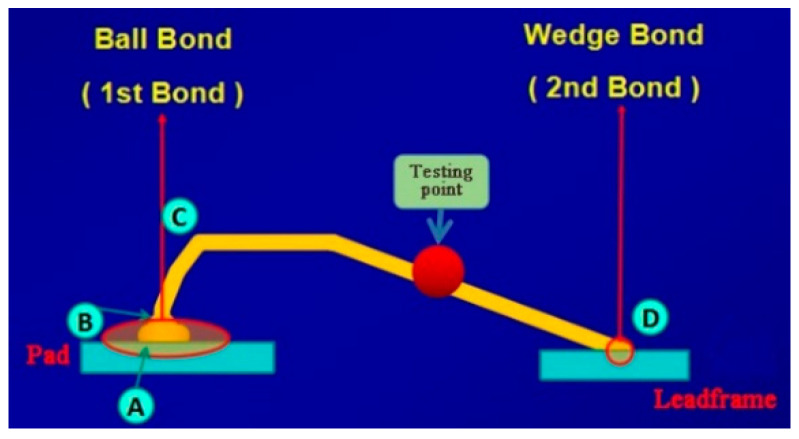
Mechanism of ball pull strength and wedge pull strength testing [27].

During the bonding process, the FAB is deformed because of the bonding power, bonding force and the heat applied, as shown in Figure 7. The extent of deformation of the FAB depends on the bonding power and bonding force. The bonded geometry of the Ag-10Au-3.6Pd alloy wire for a bonding power of 85 mW and bonding force of 0.75 N is shown in Figure 8. In this case, the bonded ball overflows, and neck cracks develop in it. This phenomenon can cause the device to short circuit and fail. On the other hand, the bonded ball is regular when the bonding power is 70 mW and the bonding force is 0.60 N, as shown in Figure 9. However, when the bonding power is 55 mW and the bonding force is 0.45 N, the bonded ball become smaller, as shown in Figure 10. The ball shear strength for different bonding parameters is shown in Figure 11. The mechanism of ball shear strength testing is shown in Figure 12. It can be seen from the figure that the ball shear strength is the lowest when the bonding power is 55 mW and the bonding force is 0.45 N. Thus, these conditions are unsuitable, as the shear strength does not meet the requirements. However, when the bonding power is 85 mW and the bonding force is 0.75 N, the ball shear strength is higher than the standard value. But, in this case, the bonded exhibits a stress concentration and can readily cause a short circuit, resulting in device failure. The ball pull strength of the bonding wire for different bonding power and force is shown in Figure 13, the mechanism of ball pull strength testing is shown in Figure 14, the broken is B. It is evident from the figure that the distribution of the ball pull strength is wide when the bonding power is 85 mW and the bonding force is 0.75 N. However, in some cases, the ball pull strength is very low, in these cases, device failure may occur. For a bonding power of 70 mW and bonding force of 0.60 N, the ball pull strength is stable and meet the requirements. On the other hand, for a bonding power of 55 mW and bonding force of 0.45 N, the distribution of the ball pull strength is wide. Finally, the ball bond is regular and the bonding shear strength is as high as the desired value when the bonding power is 70 mW and the bonding force is 0.60 N.

During bonding process, the bonding power and bonding force are applied on the bonding wire through the capillary to ensure that the bonding wire and pad are in close contact. The bonding wire and pad surface exhibit high-frequency vibrations because of the bonding power, this removes all the contaminants present on the wire and pad surface. At the same time, a large number of dislocations are produced within the bonding wire and pad. This results in the formation of fast diffusion channels between the bonding wire and substrate. For the investigated ball bonded, when the bonding power and force are 55 mW and 0.45 N, respectively, the energy delivered and pressure applied are not enough to completely remove all the contaminants and oxides present on the bonding wire and pad surface. As a result, the number of dislocations in the bonding wire and pad remain small, and the diffusion rate between the bonding wire and pad is also low. As a result, the ball shear strength is small and does not meet the requirements. However, when the bonding power and force are 85 mW and 0.75 N respectively, the FAB undergoes deformation owing to the large bonding force. This leads to the overflowing of the ball, even the formation of chips and cracks can be found. At the same time, the excessive power increases the stress in the neck and may cause it to crack. This, in turn, decreases the pull strength, as shown in Figure 13.

### 3.5. Effects of Bonding Power and Force on Wedge Bonded Strength of Ag-10Au-3.6Pd Alloy Wire

**Figure 15:**
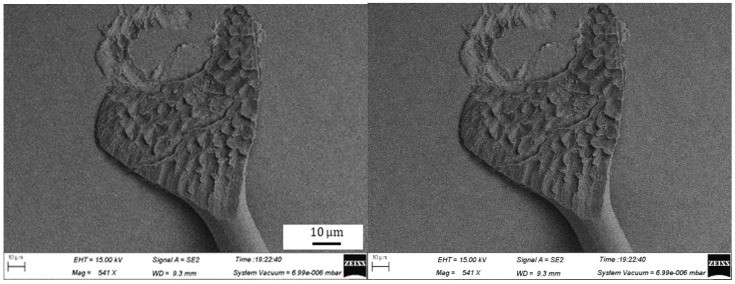
Geometry of wedge bonded formed for bonding power of 105 mW and bonding force of 1.00 N.

**Figure 16:**
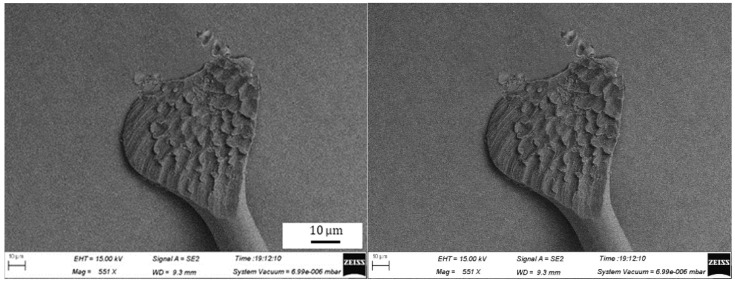
Geometry of wedge bonded formed for bonding power of 80 mW and bonding force of 0.7 N.

**Figure 17:**
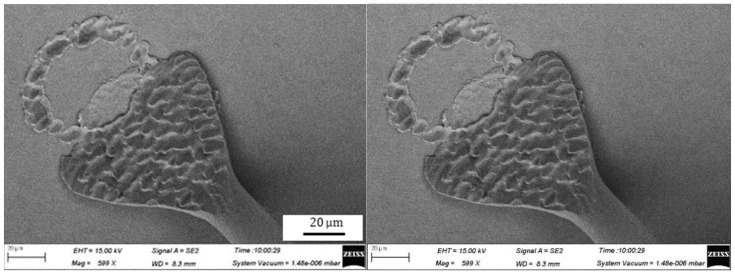
Geometry of wedge bonded formed for bonding power of 95 mW and bonding force of 0.85 N.

The geometries of wedge bonds formed using the Ag-10Au-3.6Pd alloy wire are shown in Figure 15, the bonding power and force used are 105 mW and 1.0 N, respectively. It can be seen that the extent of deformation of the fish tail is high and that the pad is also significantly deformed. The geometry of the bond formed for a bonding power of 80 mW and bonding force of 0.7 N is shown in Figure 16. It can be seen that, in this case, the fish tail is short and asymmetric. Finally, the geometry for a bonding power of 95 mW and bonding force of 0.85 N is shown in Figure 17. It can be seen that the geometry of the bond formed in this case is excellent.

Generally speaking, if the ultrasonic power is too high, the ultrasonic softening effect will become obvious. But if the ultrasonic power is too high, it will cause work hardening [28,29]. In order to have larger friction energy during bonding, larger bonding pressure is generally used. However, too high bonding pressure will limit the degree of slip between interfaces and reduce the friction energy, thus reducing the bonded strength of solder joints [30,31].

**Figure 18:**
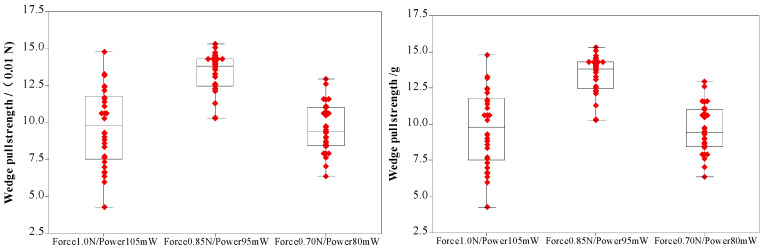
Pull strength of wedge bonded.

During the bonding process, the metal atoms diffuse rapidly because of the application of the bonding power and force, resulting in bonded formation. When the bonding power is 105 mW and bonding force is 1.00 N, the energy and contact stress between the bonding wire and pad are excessively high, because of which the pad is deformed. This decreases the bonded strength. In this case, the distribution of the wedge pull strength is wide, and in some cases, the wedge pull strength is lower than the standard, as shown in Figure 18, the mechanism of wedge pull strength testing is shown in Figure 14, the broken is D. In these cases, the device life will be decreased. In addition, the use of excessively large bonding power and force can cause significant stress concentration in the bonded area, decreasing the bond reliability. Moreover, when the values of the bonding parameters are too large, the fish tail length is reduced and the effective contact area between the bonding wire and pad becomes small and hence the bonded strength decreases. Finally, there is stress concentration in the fish tail, which can cause the bonded to break. On the other hand, a bonding power of 80 mW and bonding force of 0.70 N are insufficient to remove all the contaminants and oxides present on the wire surface and pad. In this case, the number of dislocations formed within the wire is insufficiently, then resulting in the formation of fewer diffusion channels between the wire and pad surface. As a result, the rate of diffusion between the wire and pad surface is reduced, this decreases the strength of the wedge bonded, as shown in Figure 16. For the Ag-10Au-3.6Pd alloy bonding wire, the bonded strength was found to be high for a bonding power of 95 mW and bonding force of 0.75 N.

## 4. Conclusions

In this study, the effects of different EFO and bonding parameters on the bonds formed using an Ag-10Au-3.6Pd alloy bonding wire were analyzed. The primary conclusions of the study can be summarized as follows:

(1) For the Ag-10Au-3.6Pd alloy bonding wire (diameter of 0.025 mm), for a constant EFO time, the geometry of the FAB changes from a pointed ball to an oval ball, round ball and golf ball when EFO current is increased. On the other hand, for a constant EFO current, the FAB geometry changes from a small round ball to a regular round ball and finally to a golf ball. The FAB was a regular round ball when the EFO time was 0.80 ms and EFO current was 0.030 A.
(2) For the investigated bonding wire, at an EFO current of 0.030 A, the FAB diameter and EFO time exhibited a nonlinear relationship, which could be expressed by a quadratic function.
(3) For the Ag-10Au-3.6Pd alloy bonding wire, the ball bonded is regular and the ball shear strength meets the requirements when the bonding power is 70 mW and the bonding force is 0.60 N. On the other hand, the geometry of the wedge bonded is excellent when the bonding power is 95 mW and the bonding force is 0.85 N.

## Figures and Tables

**Table 1:** Bonding parameters for Ag-10Au-3.6Pd alloy wire.

Ball Bond		Wedge Bond		Free Air Ball	
Impact Force *F_f_*/N	0.65	Bond Force *F*_1_/N	0.75	Spark Voltage/V	5000
Bond Force *F*/N	0.45/0.60/0.75	US Power *P*_1_/mW	60	Electronic Flame Off (EFO) Current/A	0.015/0.020/0.025/0.030/0.035
US Power *P*/mW	55/70/85	Bond Time *t*_1_/ms	6	EFO Time/ms	1.00/0.90/0.80/0.70/0.60
Bond Time *t*/ms	8	Bond Force *F*_2_/N	0.70/0.85/1.00	Tail Length/mm	0.15
		US Power *P*_2_/mW	80/95/105	Bonding Temperature/°C	220
		Bond Time *t*_2_/ms	6		
